# A new genus and species of octocoral with aragonite calcium-carbonate skeleton (Octocorallia, Helioporacea) from Okinawa, Japan

**DOI:** 10.3897/zookeys.511.9432

**Published:** 2015-07-02

**Authors:** Yu Miyazaki, James Davis Reimer

**Affiliations:** 1 Molecular Invertebrate Systematics and Ecology Laboratory, Graduate School of Engineering and Science, University of the Ryukyus, 1 Senbaru, Nishihara, Okinawa 903-0213, Japan; 2 Tropical Biosphere Research Center, University of the Ryukyus, 1 Senbaru, Nishihara, Okinawa 903-0213, Japan

**Keywords:** Aragonite skeleton, molecular phylogeny, new species, octocoral, relict species, taxonomy

## Abstract

A new genus and species of octocoral with a calcium-carbonate skeleton, *Nanipora kamurai* **sp. n.**, is described from a shallow coral reef in Okinawa, Japan. Contrary to most octocorals, the skeleton is composed of crystalline aragonite as in blue coral *Heliopora*. The results of molecular phylogenetic analyses of sequences of mtMutS, COI, and ITS1-5.8s-ITS2-28S region suggest *Nanipora* **gen. n.** specimens should be included in order Helioporacea. Based on morphological results compared with other Helioporacea including the genus *Epiphaxum* (family Lithotelestidae), we establish the new genus *Nanipora* within Lithotelestidae. This is the first time that a close molecular phylogenetic relationship between *Heliopora* and a related genus within Helioporacea has been revealed.

## Introduction

Octocorals (class Anthozoa, subclass Octocorallia) are sessile marine benthic organisms. Most octocorals support their body by sclerites in their tissue, or having a solid axial structure made of calcite calcium-carbonate or of protein, unlike scleractinians with a massive aragonite calcium-carbonate skeleton. The blue coral *Heliopora coerulea* (Pallas, 1766) (Helioporacea, Helioporidae) is especially peculiar as it is an octocoral with a massive aragonite calcium-carbonate skeleton similar to scleractinians.

Although *Heliopora coerulea* was long considered to be the sole member of the order Helioporacea, Bayer and Muzik (1977) described an octocoral with aragonite skeleton from a specimen in the Barbados collection of J. B. Lewis as *Lithotelesto micropora* (Bayer & Muzik, 1977) and placed the species in the family Lithotelestidae established within Helioporacea. Bayer (1979) reclassified this species as *Epiphaxum micropora*, as Lonsdale (1850: 237–324) had previously described a very similar fossil octocoral as *Epiphaxum auloporoides* Lonsdale, 1850. In total, two fossil and three extant *Epiphaxum* species, two from the Caribbean and one from the western Indian Ocean (Madagascar) (Bayer 1992; Louzet and Molodtsova 2008), have been recorded. While fossils of *Epiphaxum* have been recovered sporadically but widely from Europe, from a wide range of geological ages (Lozouet and Molodtsova 2008), extant species’ records are very rare and this genus remains enigmatic (Daly et al. 2007; McFadden et al. 2010).

In this study, we report on our examinations of unknown octocoral specimens with a calcium-carbonate skeleton from a shallow reef off Zamami Island, Okinawa, Japan. Morphology and structure of skeleton for these specimens were examined by using SEM and micro-CT. X-ray diffraction was used to determine the calcium-carbonate composition of the skeleton. Three molecular markers; mitochondrial mismatch repair protein (mtMutS), mitochondrial cytochrome c oxidase subunit 1 (COI), and the nuclear ribosomal gene complex of the 3’ end of the 18S subunit, ITS-1, 5.8S subunit, ITS-2, and the 5’ end of the 28S subunit (ITS1-5.8s-ITS2-28S) were sequenced to determine the phylogenetic placement of these specimens. Based on these specimens from Zamami Island, this octocoral is described as *Nanipora kamurai* gen. et sp. n. within the family Lithotelestidae.

## Methods

### Collection of specimens

Specimens were collected by snorkeling using a chisel and a hammer from Ama Beach, Zamami Island, Ryukyu Archipelago, Japan (26°23'N; 127°29'E) at a depth of 1 m in July 2012 (Suppl. material 1). Colonies were attached to the bottoms (=downward facing sides) of carbonate stones. Digital images were also taken in situ to record the appearance of living colonies. Specimens were fixed in 99% ethanol immediately after collection.

### Morphological analyses

**Figure 1.**
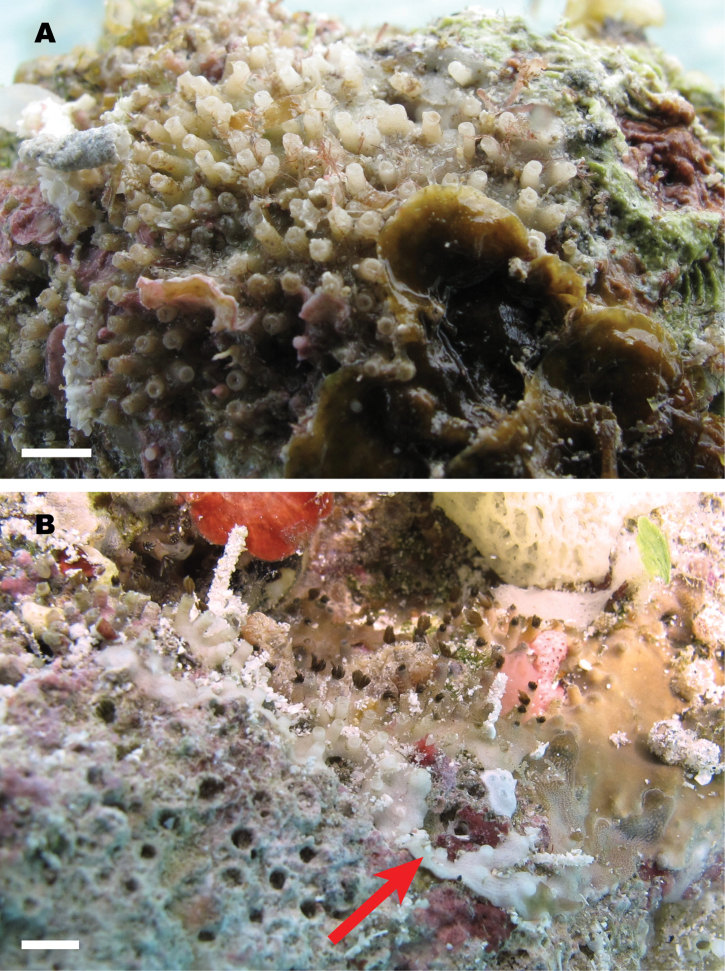
Living colony of *Nanipora kamurai* attached to the bottoms (=downward facing side) of calcium-carbonate stone at Ama Beach, Zamami Island, Okinawa, Japan, 16 July 2012. Scale bar: approximately 5 mm.

Digital images were utilized to examine the color and shape of living colonies and polyps (Fig. 1). Skeletons were soaked in household bleach containing sodium hypochlorite for 15–20 min, followed by rinsing with distilled water and air-drying, to remove soft tissues. Skeletal specimens were stuck to aluminum specimen mounts by carbon double-faced tape, then examined and imaged with a scanning electron microscope (SEM) VE-8800 (Keyence, Osaka, Japan). CT images of ethanol-preserved specimens were taken with micro CT (in vivo micro X-ray CT system R_mCT2, Rigaku, Tokyo, Japan) and examined with the DICOM imaging application OsiriX v. 5.9 32-bit (Rosset et al. 2004) to investigate the structure of the skeleton in a non-invasive manner. By using this application, areas of particular CT-value in organisms can be visualized: e.g. showing skeleton (high CT-value) and hiding soft tissue (low CT-value), or vice versa. To check for the presence of sclerites, small pieces of EtOH preserved colonies were dissolved with household bleach on a well-slide and examined with an optical microscope. Additionally, several polyps were dissolved on carbon double-faced tape. Deposits from washing solution of whole colonies were retrieved and carefully rinsed, followed by mounting on carbon double-faced tape. These specimens were examined by SEM. X-ray diffraction analyses were performed with an X-ray diffractometer RINT Ultima/PC (Rigaku, Tokyo, Japan) at the Instrumental Research Center, University of the Ryukyus, to examine the crystal forms of calcium carbonate (aragonite/calcite) of the skeleton. Skeletal specimens were smashed to powder, mounted on well-washed glass slides and analyzed using Cu Kα radiation (40 kV, 30 mA), scanning between a 2θ angle ranging from 25° to 37° at 0.02° steps. The calcite/aragonite component (%) of samples was determined by comparing with data of standard samples of 100% calcite/aragonite. For comparison, a sample skeleton of the blue coral *Heliopora coerulea* was prepared and analyzed in the same manner.

### DNA extraction and PCR amplification

DNA was extracted from tentacles and anthocodial tissue of ethanol-preserved samples by a guanidine extraction protocol following Sinniger et al. (2009). PCR amplifications were performed using HotStarTaq DNA polymerase (Qiagen, Tokyo, Japan) according to the manufacturer’s instructions. The mitochondrial mismatch repair protein (mtMutS) was amplified by semi-nested PCR following the procedure of McFadden et al. (2006), as no visible PCR product was yielded by a single PCR reaction. First, primers ND42599F (5’-GCCATTATGGTTAACTATTAC-3’) (France and Hoover 2002) and Mut-3458R (5’-TSGAGCAAAAGCCACTCC-3’) (Sánchez et al. 2003) were used to amplify the 5’ end of mtMutS, and then a second PCR reaction was run using internal forward primer ND42625F (5’-TACGTG GYACAATTGCTG-3’) (Lepard 2003) and reverse primer Mut-3458R. Both PCR reactions were performed under the following conditions: 15 min at 94 °C; 35 cycles of 1.5 min at 94 °C, 1.5 min at 58 °C, and 1 min at 72 °C; and a final extension of 5 min at 72 °C. Mitochondrial cytochrome oxidase subunit I (COI) was amplified by the primers COII-8068 (5’-CCATAACAGGACTAGCAGCATC-3’) (McFadden et al. 2004) and COI-OCTr (5’-ATCATAGCATAGACCATACC-3’) (France and Hoover 2002) under the following conditions: 5 min at 95 °C; 35 cycles of 1 min at 94 °C, 1 min at 40 °C, and 1.5 min at 72 °C; and then a final extension of 7 min at 72 °C. The nuclear ribosomal gene complex of the 3’ end of the 18S subunit, internal transcribed spacer 1 (ITS-1), 5.8S subunit, ITS-2, and the 5’ end of the 28S subunit was amplified by the primers 1s (5’-GGTACCCTTTGTACACACCGCCCGTCGCT-3’) and 2ss (5’-GCTTTGGGCTGCAGTCCCAAGCAACCCGACTC-3’) (Chen et al. 1996) under the following conditions: 15 min at 95 °C; 35 cycles of 0.5 min at 94 °C, 1 min at 52 °C, and 1.5 min at 72 °C; and a final extension of 5 min at 72 °C. Amplified products were visualized with 1.0% agarose gel electrophoresis. Positive PCR products were cleaned up by Exonuclease I and Shrimp Alkaline Phosphatase (Takara, Shiga, Japan) before sequencing.

### Sequence analyses

Sequencing was performed by Fasmac (Kanagawa, Japan). Cycle sequencing was performed in both directions using the forward and reverse primers separately with BigDye® Terminator v3.1 Cycle Sequencing Kit (Applied Biosystems) under reaction conditions according to the manufacturer’s instructions. Reaction products were analyzed on an ABI PRISM 3700 DNA Analyzer (Applied Biosystems). The sequences were analyzed by 4Peaks Version 1.7.2 software (mekentosj.com, Amsterdam, Netherlands).

By using Se-AL v2.0a11 software (Rambaut 2002), the nucleotide sequences of mtMutS, COI, and ITS1-5.8s-ITS2-28S from specimens obtained in the present study were separately aligned with sequences of *Heliopora* and other octocoral species retrieved from GenBank (Suppl. material). The alignments were checked by eye and manually edited to remove any ambiguous sites (e.g. double peaks) before phylogenetic analyses. For each alignment, none or only one to two base pairs were edited in this manner. Consequently, three aligned data sets were generated: 1) 861 sites of 33 sequences (mtMutS), 2) 735 sites of 12 sequences (COI), and 3) 697 sites of 5 sequences (ITS1-5.8s-ITS2-28S). The alignment data are available on request from the corresponding author. Additional octocoral sequences retrieved from GenBank are shown in Supplementary Table 2.

### Phylogenetic analyses

Maximum-likelihood (ML) analyses with PhyML Online (Guindon and Gascuel 2003) of these datasets were independently performed using input trees generated by BIONJ (Gascuel 1997) with the general time reversible (GTR) model. PhyML bootstrap trees (1000 replicates) were constructed using the same parameters as the individual ML trees. Genetic distances were calculated using Kimura’s two-parameter model (Kimura 1980).

Bayesian trees were reconstructed by running the program MrBayes 3.1.2 (Ronquist and Huelsenbeck 2003) within the program Geneious version 8.0.4 (Restricted) created by Biomatters (available from http://www.geneious.com/). One cold and three heated Markov chain Monte Carlo (MCMC) chains with default-chain temperatures were run for 1,000,000 generations, sampling log-likelihoods (InLs), and trees at 1000-generation intervals (1,000 InLs and trees were saved during MCMC). The first 100,000 generations of all runs were discarded as “burn-in” for the dataset and remaining 900 trees were used to obtain posterior probabilities and branch-length estimates, respectively. Neighbor-joining (NJ) trees were also reconstructed by using CLC Free Workbench 4 software (CLCbio.com, Aarhus North, Denmark) (1000 replicates).

## Systematics

### Class ANTHOZOA Ehrenberg, 1831 Subclass OCTOCORALLIA Haeckel, 1866 Order HELIOPORACEA Bock, 1938

#### Family Lithotelestidae

Taxon classificationAnimaliaHelioporacea

Bayer & Muzik, 1977

##### Type genus.

*Lithotelesto* Bayer & Muzik, 1977 (junior synonym of *Epiphaxum* Lonsdale, 1850).

##### Diagnosis

(after Bayer and Muzik 1977; Lozouet and Molodtsova 2008, revised). Helioporacean octocoral with growth form of encrusting, stoloniferous or upright sparsely branched stems. Whole colony rigid with internal skeleton of crystalline aragonite. Polyps fully retractile. Sclerites may be present or absent; if present, capstans and crosses in form, composed of calcite.

#### Genus Nanipora gen. n.

Taxon classificationAnimaliaHelioporaceaLithotelestidae

http://zoobank.org/283000C2-355E-4302-A44F-94CB19202538

##### Type species.

*Nanipora kamurai* sp. n., here designated.

##### Diagnosis.

Encrusting, partly stoloniferous colony with cylindrical calyces up to 5 mm tall, attached to hard substratum. Polyps monomorphic and retractile. Coenenchyme and calyces rigid with internal skeleton, not composed with fused sclerites but of unitary crystalline aragonite. Reticulate pattern on whole colony’s surface, made of tiny pores on the surface. Top of the calyx serrated, with 16–20 indentations. Longitudinal cavities in the calicular wall, connecting to solenial canals in the base of the colony. Interior of calyx smooth, lacking septae. Surface of calyces occasionally wrinkled. Completely lacks sclerites. Living colonies ivory or pale brown. Skeleton colourless. Azooxanthellate.

##### Etymology.

Named from the Japanese ‘nani’ plus latin ‘pora’: ‘nani’ means ‘what is this?’, as the genus is highly unusual in having an aragonitic skeleton; ‘pora’ is originally meaning of ‘pore’, name used for many anthozoan (especially scleractinian) species with porous skeleton. Gender is feminine.

#### Nanipora kamurai sp. n.

Taxon classificationAnimaliaHelioporaceaLithotelestidae

http://zoobank.org/98A4C103-57A8-469B-A157-81B088EDC717

Figs 1 , 2 , 3 , 4 , 5 , 6 , 7 , 8 , 9 , 10 , 11

##### Type material.

Holotype: NSMT-Co1562, Ama Beach, Zamami, Okinawa, JAPAN (26°13.31'N; 127°17.28'E), 1 m depth, collected by Yu Miyazaki (Y.M.), 16 July 2012, fixed in 99% EtOH, deposited in National Museum of Nature and Science, Tokyo, Japan (NSMT). GenBank accession numbers: mtMutS, KP195280; mt COI, KP195281; ITS1-5.8s-ITS2-28S, KP195282; Paratype 1: Specimen number RMNH 41731. Ama Beach, Zamami, Okinawa, JAPAN (26°23'N; 127°29'E), 1 m depth, collected by Yu Miyazaki (Y.M.), 16 July 2012, fixed in 99% EtOH, deposited in Naturalis Biodiversity Center, Leiden, the Netherlands (RMNH). Paratype 2: USNM 1231377, Ama Beach, Zamami, Okinawa, JAPAN (26°23'N; 127°29'E), 1 m depth, collected by Yu Miyazaki (Y.M.), 16 July 2012, fixed in 99% EtOH, deposited in National Museum of Natural History, Smithsonian Institution, Washington, D.C., USA (USNM); Other materials. Specimen number MISE-MY-120715. Ama Beach, Zamami, Okinawa, JAPAN (26°23'N; 127°29'E), 1 m depth, collected by Yu Miyazaki (Y.M.), 15 July 2012, fixed in 99% EtOH.

##### Description.

The holotype colony is encrusting (Fig. 1A), attached to the bottom (=downward facing side) of carbonate stone of dimensions 80 × 50 × 50 mm. Colony occasionally with thin stolons (2–3 mm in width, less than 1 mm thick) growing over irregular surface (Fig. 1B, arrow). The polyps of holotype colony are completely withdrawn into calyces after fixation.

**Figure 2.**
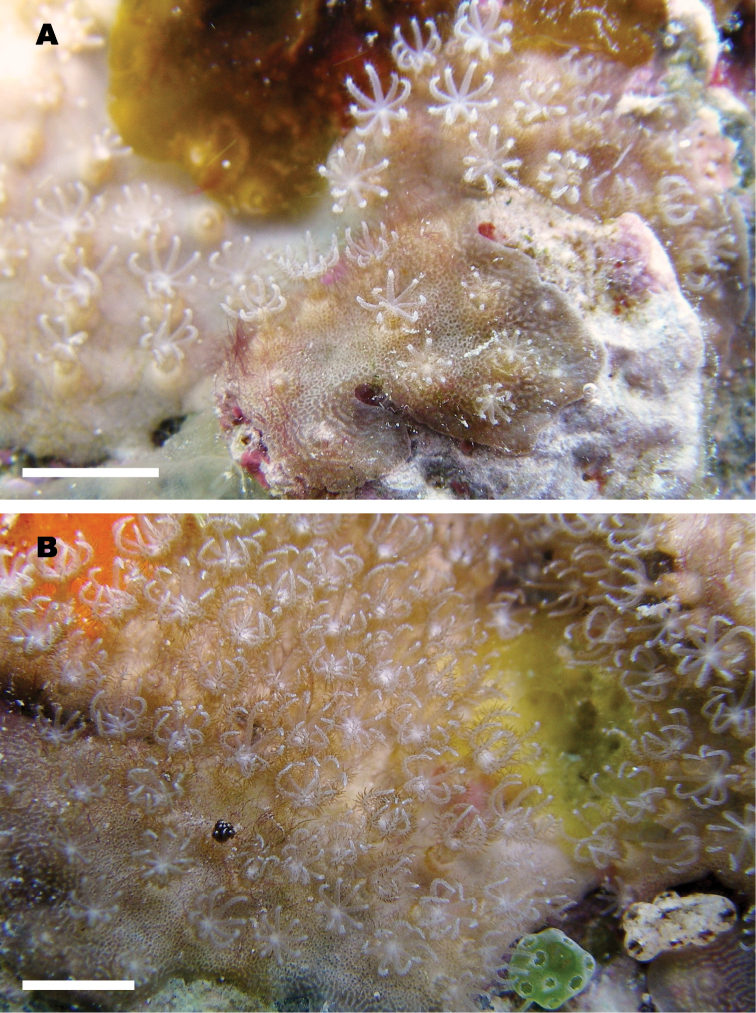
In situ colony of *Nanipora kamurai* with expanded polyps at Ama Beach, Zamami Island, Okinawa, Japan, 16 July 2012. **A** growing edge of the colony **B** middle portion of the colony. Scale bar: approximately 5 mm.

Tentacles are 3–4 mm long, with fine but distinct pinnules (Fig. 2A and B). Anthocodiae are fully retractile within calyces. Coenenchyme is thin (up to 3 mm, less than 1 mm in most portions). Both coenenchyme and calyces are rigid with internal skeletons.

**Figure 3.**
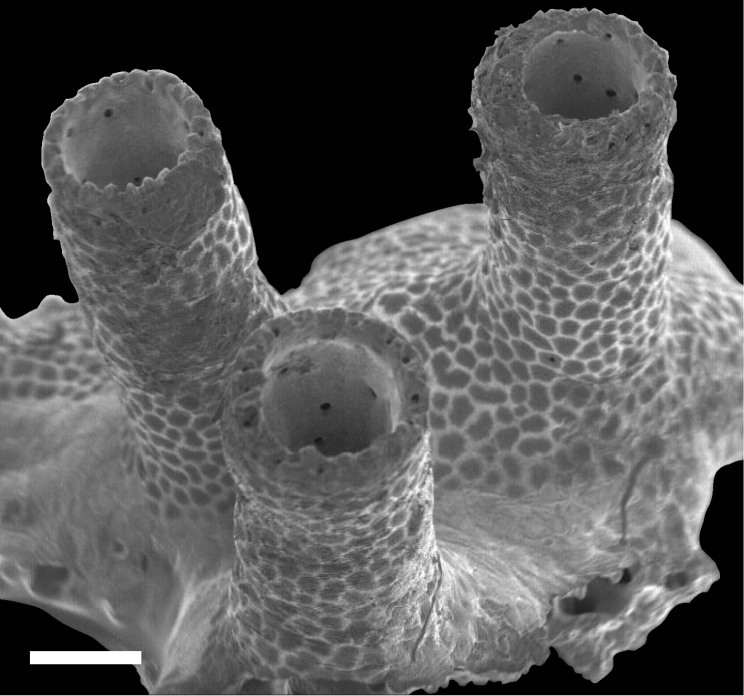
Scanning electro-microscope (SEM) image for skeleton of *Nanipora kamurai* colony. Scale bar: 0.5 mm.

**Figure 4.**
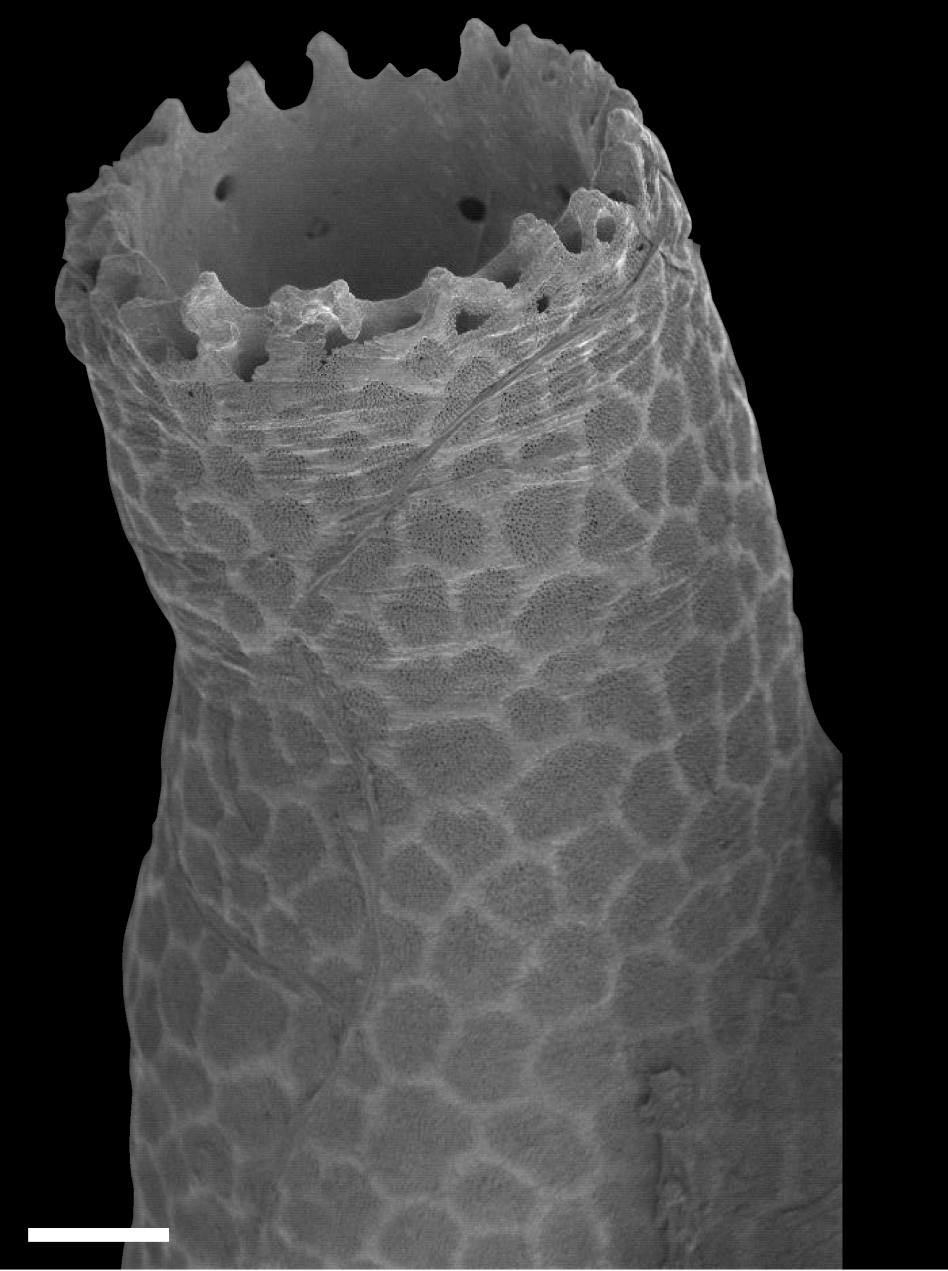
Calyx of *Nanipora kamurai*. Reticulate pattern and wrinkles are shown. Scale bar: 0.2 mm.

**Figure 5.**
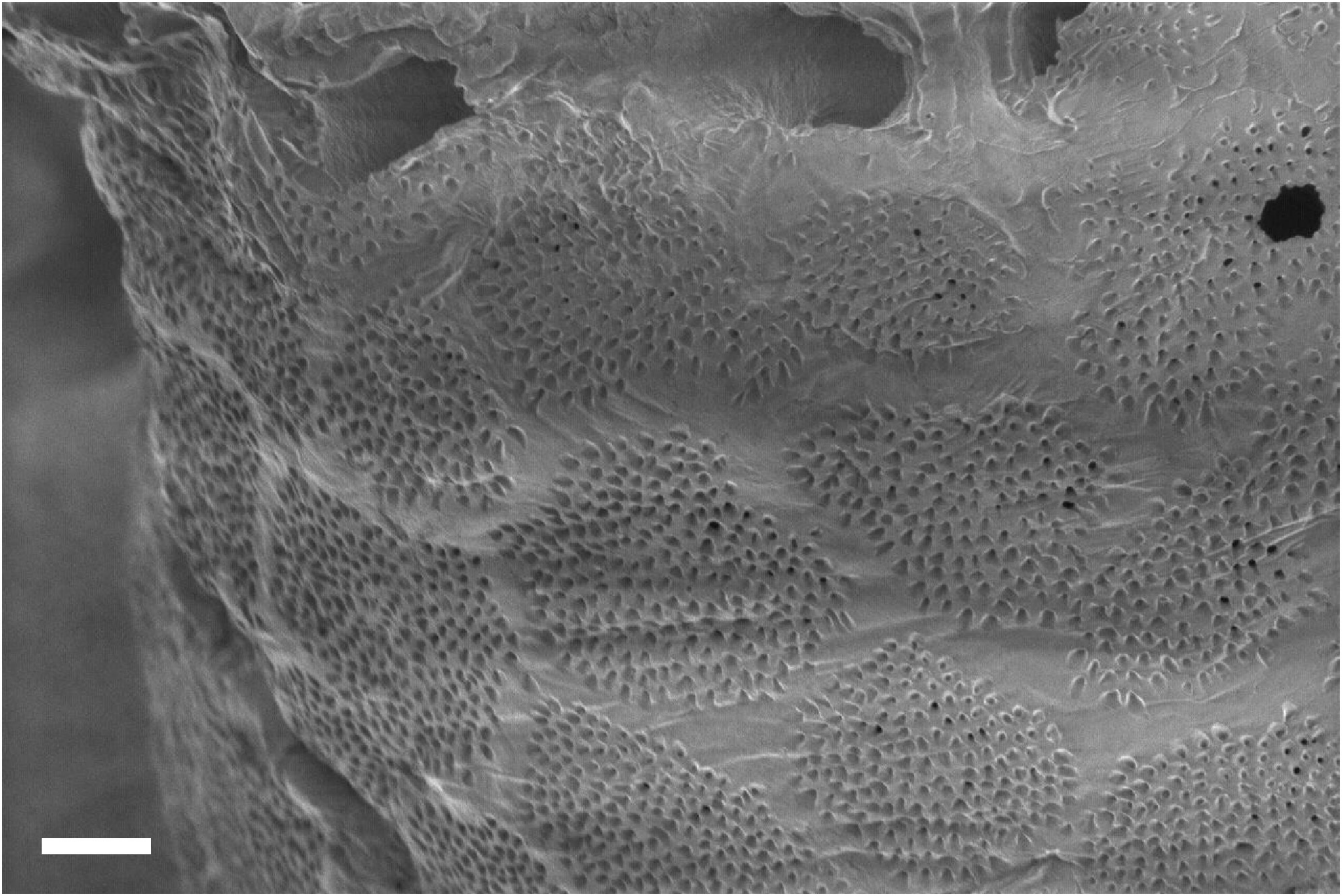
Surface of the calyx. Reticulate pattern made by numerous tiny pores. Scale bar: 0.04 mm.

**Figure 6.**
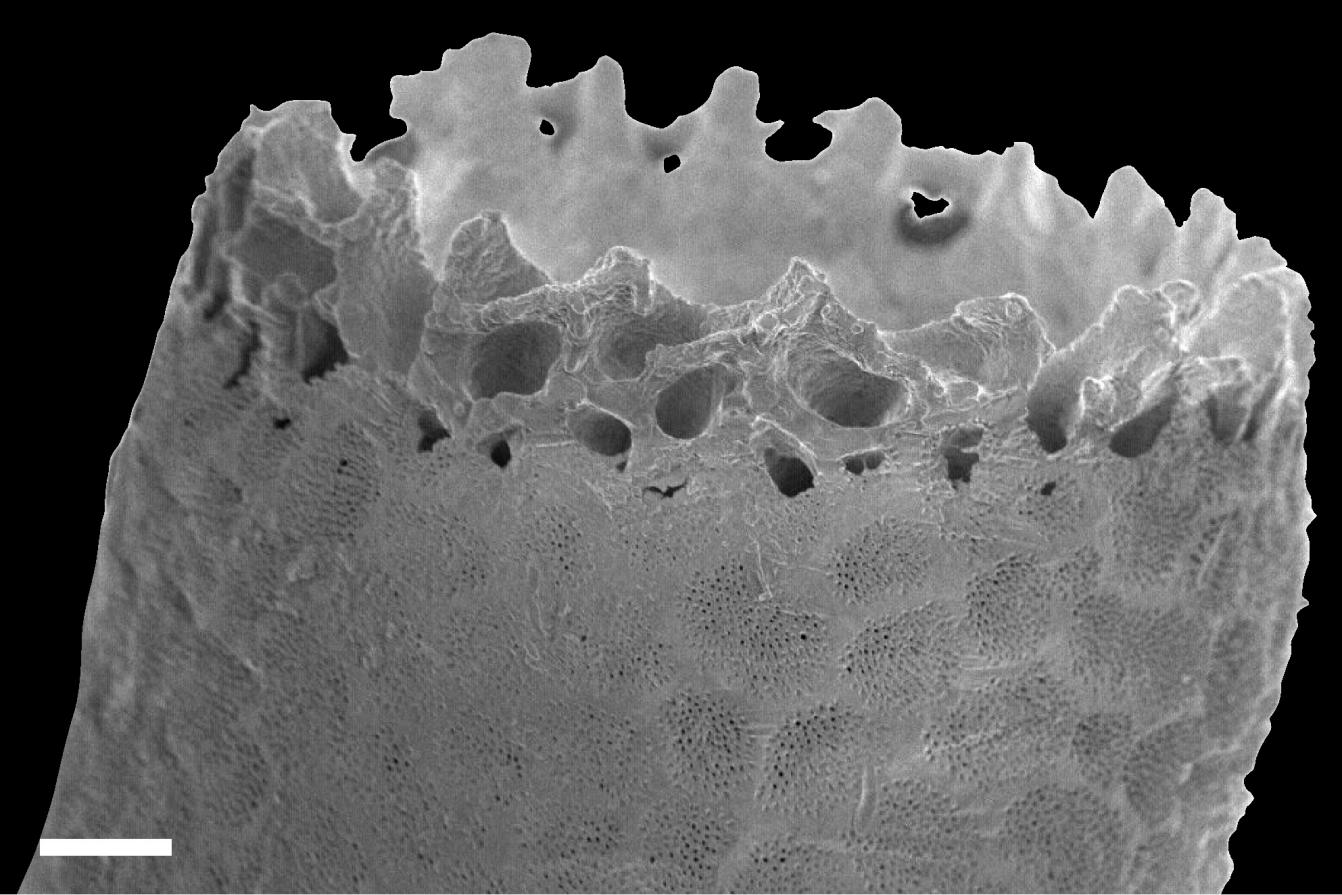
Indentations seen on the top of the calyx of *Nanipora kamurai*. Scale bar: 0.1 mm.

**Figure 7.**
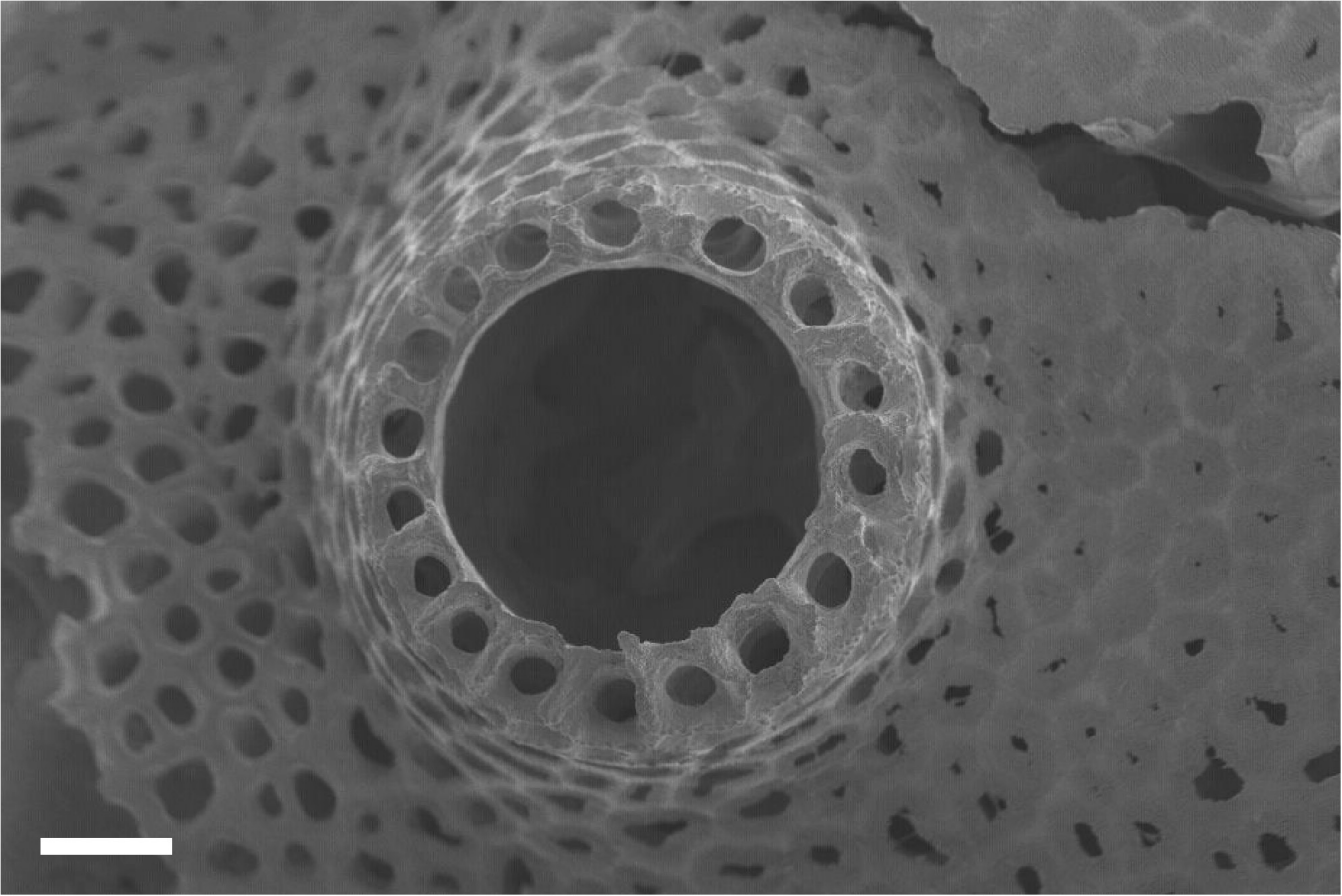
Calyx of *Nanipora kamurai* seen from above. Cavities extend in a longitudinal direction down through the calyx are shown. Scale bar: 0.2 mm.

Overall shape of the skeleton is virtually the same as the external shape of living colonies (Fig. 3). Calyces are cylindrical, up to 1 mm across; up to 5 mm in height, perforated by randomly distributed pores up to 50 μm in diameter (Fig. 4). The surface of the skeletal calyx is occasionally wrinkled (Fig. 4, 5). The top of the calyces are serrated, with usually 16, but as many as 20 indentations (Fig. 6). Inside of the calyces is simple and tubular, lacking any structures such as septae. Calicular walls are 0.08–0.1 mm thick at the apical end and gradually became thicker going down towards the proximal portion, where thicknesses reached up to 0.2 mm. In the calicular walls, from distal to proximal portions, 12–20 cavities up to 0.05 mm diameter pass through (Fig. 7). These cavities are often discontinuous, converged or branched.

**Figure 8.**
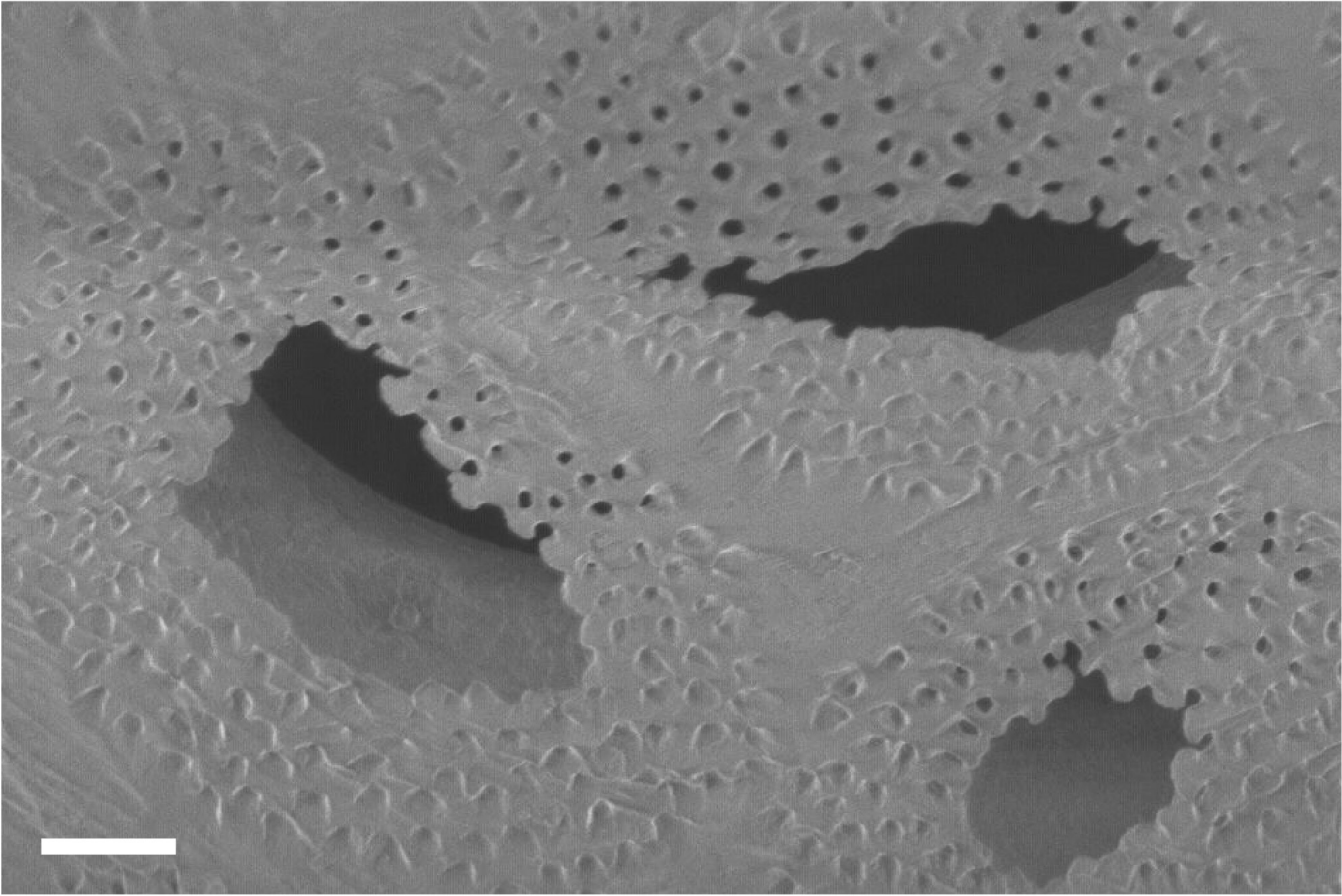
Surface calcium carbonate of the darker portions. Beneath, large holes (up to 200 μm) are shown. Scale bar: 0.02 mm.

**Figure 9.**
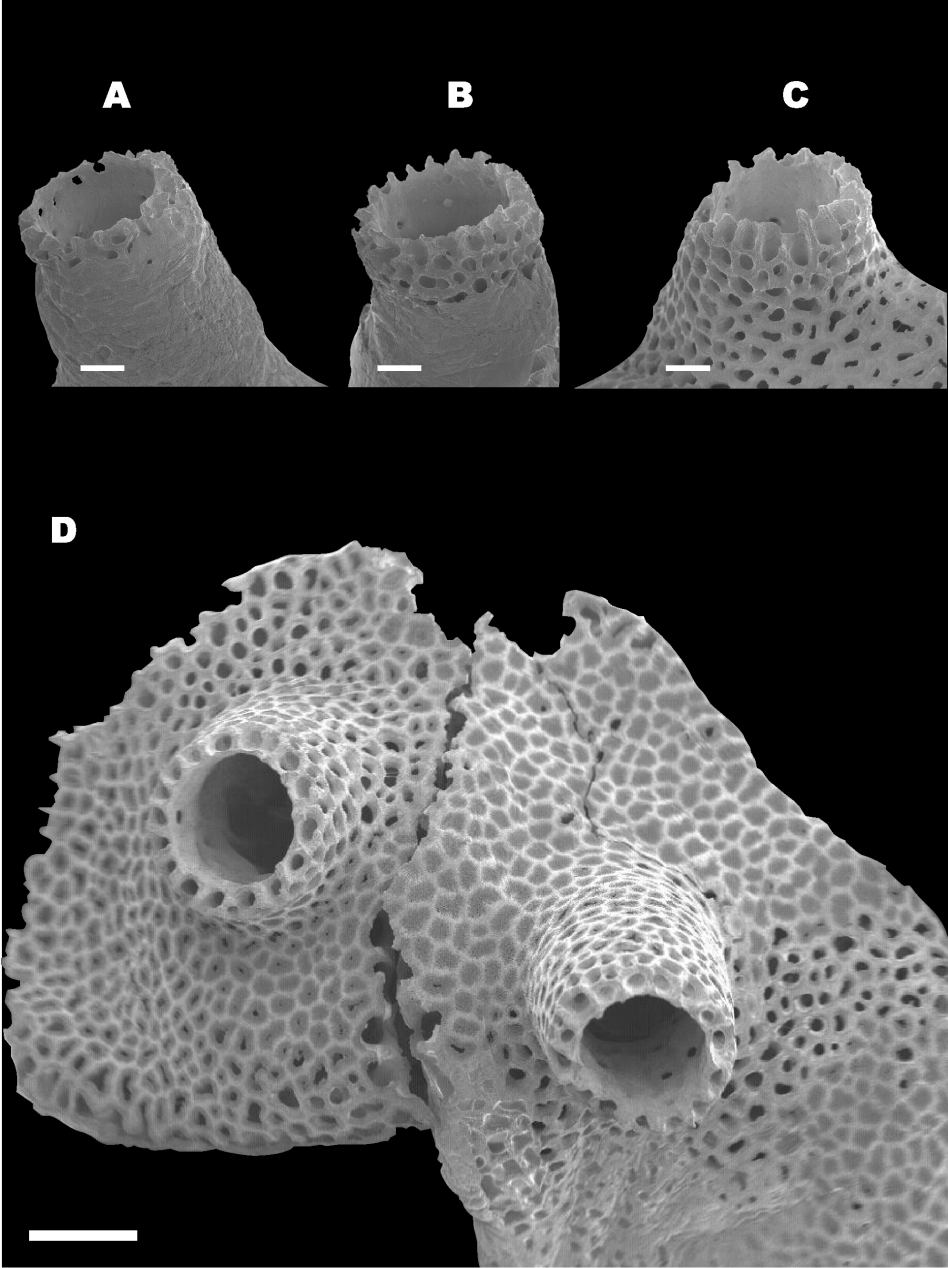
**A–C** Different surface calcium carbonate coverage of three calyces (**A>B>C**) **D** Skeleton of younger and marginal part of the colony, partly lacks surface cover. Scale bar: 0.2 mm (**A**), 0.5 mm (**B**).

**Figure 10.**
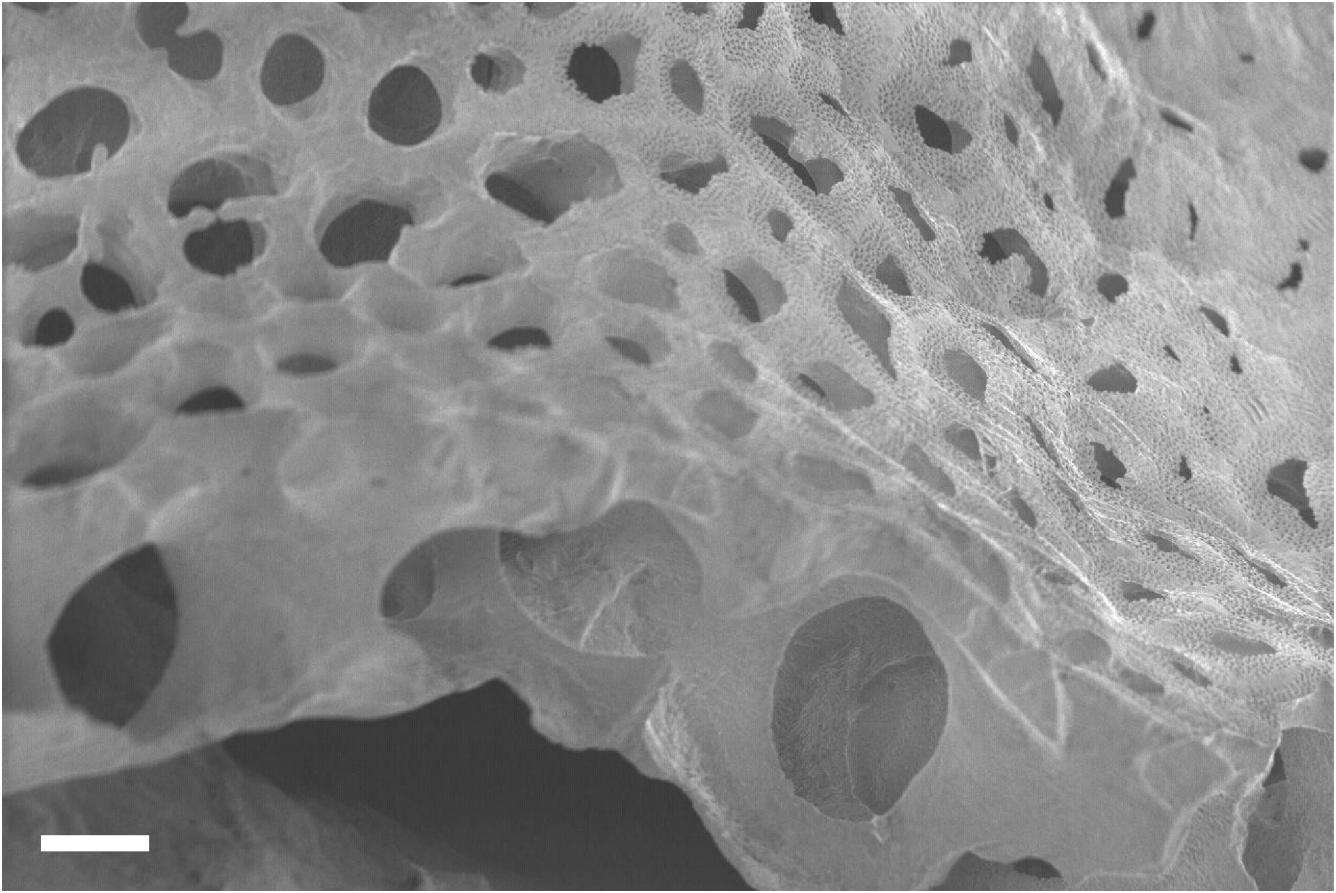
Cavities seen in cross sectioned coenenchymal skeleton. Scale bar: 0.1 mm.

**Figure 11.**
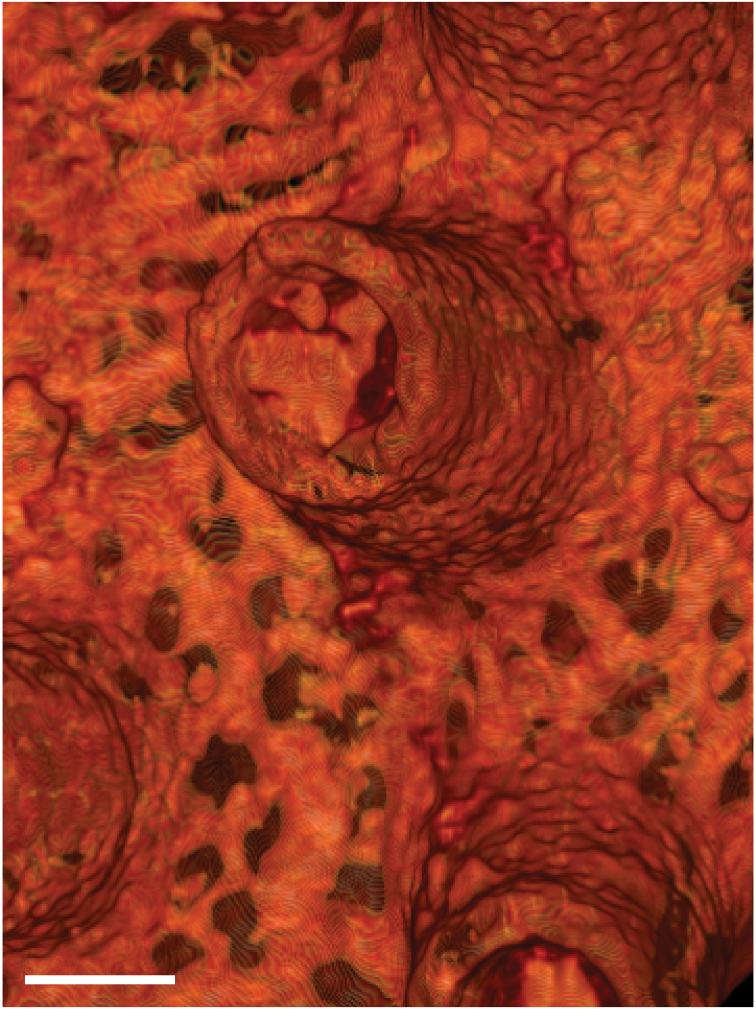
3D CT image of soft tissue. Solenial tubes forming network are shown. Scale bar: 0.5 mm.

The whole skeleton has a reticulate pattern on the surface (Fig. 3, 4). This pattern is made by numerous tiny pores (up to 5 μm in diameter, Fig. 5); darker parts with pores, and lighter parts without pores. The surface calcium carbonate of the darker portions is very thin like a sheet, compared to the part without pores (Fig. 8). Growing edges of colonies and tops of calyces tend to lack such calcium-carbonate sheets and therefore the surface of these regions has holes (up to 200 μm, Fig. 9A–D). In the cross sections of the coenenchymal skeleton, cavities 0.1–0.2 mm in diameter are observed (Fig. 10). These cavities house solenia, connecting the gastric cavities of polyps and composing solenial network (Fig. 11, 3-dimensional CT images of soft tissue). Lacks sclerites. Azooxanthellate.

**Colour.** Living colony is pale brown or ivory (Fig. 1A and B). Whole polyps are pale brown, but shrunken tentacles appear dark brown (Fig. 1B). Skeleton is colourless.

##### Etymology.

Named after Hidefumi Kamura, a great jazz pianist who has continued playing classic style be-bop jazz from when Okinawa was under the rule of U.S. forces, and who can now be considered as a ‘relict’ classical be-bop jazz musician.

##### Habitat.

*Nanipora kamurai* colonies are found on the bottoms (=downward facing sides) of carbonate stones on a sandy shallow beach at 1–1.5 m depths with very clear water. For now known only from Ama Beach, Zamami Island, Okinawa, Japan.

##### Comparison with other species.

General morphology of *Nanipora kamurai* is quite similar to *Epiphaxum* Lonsdale, 1850. Unlike *Epiphaxum* species, presence of sclerites not observed by any means in any portion of specimen in this study. Secondary daughter calyces, such as seen in *Primnoa gracilis* Nielsen, 1925 (=*Epiphaxum auloporoides*) and Verrill’s original drawing of *Lithotelesto micropora* Bayer & Muzik, 1977 (=*Epiphaxum micropora*), are not observed. Pores perforating calicular walls of *Nanipora kamurai* are distributed irregularly, not aligned in a line or in a row as seen in *Epiphaxum* species (Bayer and Muzik 1977; Bayer 1992; Lozouet and Molodtsova 2008). Neither an octagonal outline in the cross sections of calyces, such as seen in *Epiphaxum breve* Bayer, 1992, nor sclerosepta as seen in *Epiphaxum septifer*, are observed.

##### Skeleton.

Examination of SEM images clearly showed the rigid skeleton of this species was not formed by fused sclerites as in *Tubipora*, but made of unitary calcium carbonate as in *Heliopora*. X-ray diffraction analyses revealed this skeleton was composed of 96% aragonite and 4% low-Mg calcite. Inclusion of traces of calcite may be contamination from calcareous algae attached to the surface of the colony. The skeleton of blue coral *Heliopora coerulea*, analyzed for comparison, was 100% aragonite.

##### Molecular phylogeny.

**Figure 12.**
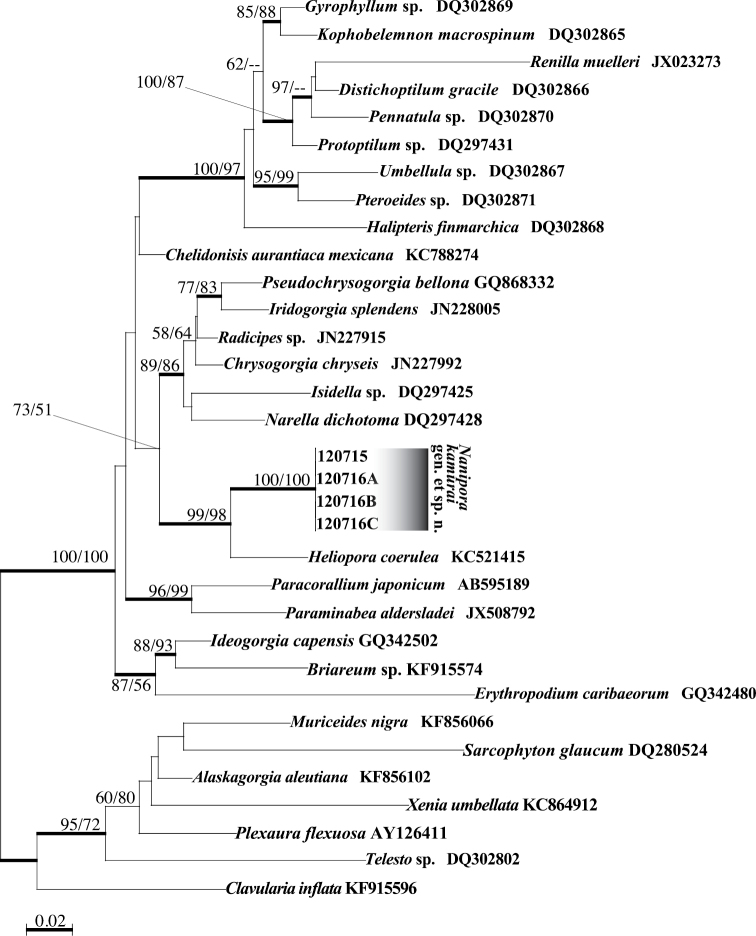
Maximum likelihood tree for mtMutS sequences. Values at branches represent ML and NJ bootstrap probabilities, respectively (>50%). Bold lines represent branches with very high support in Bayesian analyses (>0.95). Sequences without accession numbers were newly obtained in this study.

The four specimens of *Nanipora kamurai* in this study had completely identical mtMutS, COI and ITS1-5.8s-ITS2-28S sequences. In the ML trees for mtMutS (Fig. 12) and COI (not shown) alignments, the sequences of *Nanipora kamurai* in this study and *Heliopora coerulea* formed a strongly supported clade (ML=99% for mtMutS; ML=90% for COI) to the exclusion of all other octocoral sequences, and sequences of the new specimens formed a subclade clearly different from *Heliopora* (ML=100% in mtMutS, ML=100% in COI). p-distances between *Nanipora kamurai* and *Heliopora coerulea* were 0.053 (mtMutS) and 0.034 (COI), while distances between *Nanipora kamurai* and other soft corals included in phylogenetic analyses were at least >0.061 (mtMutS) and >0.042 (COI). ITS1-5.8s-ITS2-28S sequences of the unknown octocoral and *Heliopora coerulea* could be aligned together, but they could not be accurately aligned with any other known octocoral ITS1-5.8s-ITS2-28S sequences due to sequence divergence. The nuclear ITS1-5.8s-ITS2-28S region sequence of *Nanipora kamurai* had 94 nucleotide differences from *Heliopora coerulea* sequences over 697 nucleotides (=13.5% variation).

##### Remarks.

The distribution of *Nanipora* gen. n. is currently known from only one site in Okinawa. Although the lack of reports may result from their tiny size and cryptic habitat, considering the sporadic distribution and extraordinary rarity of related *Epiphaxum* spp., *Nanipora kamurai* may also be a relict species surviving with a very limited distribution. This species is one of the few exceptional species with an aragonite calcium-carbonate skeleton among Octocorallia.

##### Common Japanese name.

Zamami-ishi-hanagoke.

## Discussion

### Phylogenetic status of *Nanipora kamurai* in Helioporacea

Results of phylogenetic analyses apparently show that *Nanipora kamurai* is more closely related to *Heliopora coerulea* than to other groups of octocorals. The genetic distance between *Nanipora* and *Heliopora* as shown in branch lengths in the mtMutS tree (Fig. 12) is comparable to what is typically seen among different families of octocorals (e.g. McFadden et al. 2006) (see also Suppl. materials 3, 4). This is the first time the existence of an extant relative of *Heliopora coerulea* has been confirmed using molecular phylogenetic analyses, as no phylogenetic research on *Epiphaxum* spp. has yet been conducted.

### Taxonomic relationship between *Nanipora kamurai* and *Epiphaxum*

Lonsdale (1850), who described the fossil genus *Epiphaxum* from Chalk (Upper Cretaceous) in Sussex, United Kingdom, felt that *Epiphaxum* was not related to any extant family. According to Lozouet and Molodtsova (2008), *Epiphaxum* was later placed by Voigt (1958) into Clavulariidae, which had been defined by the presence of cylindrical anthosteles and connecting stolons. Finally, Bayer and Muzik (1977) placed this genus in the order Helioporacea by examining the type of calcium-carbonate present in skeletons.

The general colony shape of *Nanipora kamurai* closely resembles encrusting and stoloniferous species of *Epiphaxum* (Bayer, 1992). As well, the basic structure of the skeleton (simple and tubular calyx, indentations on the top of the calyx) is common among *Nanipora* and *Epiphaxum* spp. However, longitudinal grooves on the surface of calyces’ skeleton, common to every *Epiphaxum* spp. (see Bayer and Muzik 1977; Bayer 1992; Lozouet and Molodtsova 2008), were not observed in *Nanipora kamurai*. Instead, the entire surface of the *Nanipora* skeleton is covered by a reticulate pattern (Fig. 4). The various descriptions of genus *Epiphaxum* mention possession of calcite calcium-carbonate sclerites (Bayer and Muzik 1977; Bayer 1992; Lozouet and Molodtsova 2008), although actually in some species of *Epiphaxum* the presence of sclerites has not been confirmed due to the absence of soft-tissue in the holotype specimen (*Epiphaxum septifer* Bayer, 1992), or due to the type specimen being fossilized (*Epiphaxum arbuscula* Bayer, 1992) (Bayer 1992; Lozouet and Molodtsova 2008). The stoloniferous colony is one of the simplest colony morphologies in Octocorallia and is found among many unrelated groups of octocorals, but many other morphological characters apparently suggest the close relatedness of *Nanipora kamurai* and *Epiphaxum*. In particular, considering the complete lack of sclerites, placement of *Nanipora kamurai* in a new genus inside Lithotelestidae is much more appropriate than a major modification of genus *Epiphaxum* and inclusion of *Nanipora kamurai* within *Epiphaxum*. Molecular phylogenetic data for *Epiphaxum* spp. are necessary to construct the complete phylogeny of Helioporacea.

### Taxonomic status of order Helioporacea

The blue coral *Heriopora coerulea*, the sole member of Helioporidae, has been considered to be extraordinarily distinct among octocorals. When Bayer (1981) reorganized the entire subclass Octocorallia into only three orders, one order was Helioporacea. However, McFadden et al. (2006) showed that *Heliopora coerulea* sequences were placed within a large Calcaxonia–Pennatulacea clade. In this study, similar to McFadden et al. (2006), *Heliopora coerulea* and *Nanipora kamurai* sequences fell into the same sub-major clade of Octocorallia. However, although no recent molecular study supports the phylogenetic distinctiveness of Helioporacea as an order within subclass Octocorallia, the order Helioporacea is still used. The taxonomic status of this order needs to be re-examined with careful morphological and molecular phylogenetic comparisons. The discovery and confirmation of the phylogenetic relationship of *Nanipora kamurai* to Helioporacea in this study should contribute to this reassessment.

### Geographic distribution and the origin of *Nanipora kamurai*

Extant *Heliopora coerulea* is restricted to the Indo-Pacific, and extant species of *Epiphaxum* are found from the Caribbean and the western Indian Ocean (Madagascar), while fossil species of both genera have been found sporadically but widely from Europe. So far, *Nanipora* has only been found in Zamami Island, Okinawa, Japan, although surveys are needed to confirm its exact distribution. As indicated in Lozouet and Molodtsova (2008), recent *Heliopora coerulea* is considered to be a relict of fossil *Heliopora* species distributed throughout the Tethys Ocean. Considering the morphological characters of these genera, we can hypothesize that Helioporidae branched first from a common ancestor, subsequently followed by the division of *Epiphaxum* and *Nanipora*, although the geographical timing of this split is unknown. As pointed out by Lozouet and Molodtsova (2008), the discontinuous distribution of *Epiphaxum* across the Pacific and the Atlantic implies that at least *Epiphaxum* already existed before the closure of the Tethyan connection. Additionally, the morphological distinctiveness of *Nanipora* strongly supports the hypothesis that *Nanipora* and *Epiphaxum* radiated before the disjunction of the Atlantic and the Pacific, rather than *Nanipora* radiating from Indo-West Pacific *Epiphaxum*. Molecular phylogenetic analyses of Atlantic and Pacific *Epiphaxum* and corroborated studies with paleontology should help confirm the evolutionary history of this unique group.

### Cryptic habitat of *Epiphaxum* and *Nanipora*

Detailed habitat information of extant species of *Epiphaxum* is unknown as all known living specimens were obtained by dredging or trawling (Bayer and Muzik 1977; Bayer 1992). Lozouet and Molodtsova (2008) indicted fossil species of *Epiphaxum* (*Epiphaxum arbuscula*) were strongly related to submarine canyons, which also included fossil fauna found in muddy sea-floor at depths corresponding to the outer continental shelf or upper slope, submarine cave environments, and rocky circa-littoral cliffs. Considering this information together with the cryptic habitat of *Nanipora kamurai* (bottom side of carbonate stones), *Epiphaxum* and *Nanipora* appear to not reside in shallow well-lit subtropical and tropical environments like *Heliopora*, but instead in shaded or cryptic environments. Detailed surveys of such cryptic environments may lead to the discovery of other unknown *Epiphaxum* or *Nanipora* species.

### Importance of cryptic fauna in Octocorallia

It is astonishing that a unique, relict species such as *Nanipora kamurai* was found from shallow waters. Generally, relict species are thought to be most commonly found in stable and undisturbed environments, such as abyssal waters, as demonstrated by the discovery of Coelacanthiformes in Africa and Indonesia (Smith 1939; Erdmann et al. 1998). Although not from abyssal depths, extant *Epiphaxum* spp. were also found from quite deep habitats (*Epiphaxum micropora*: 50–400 m, *Epiphaxum breve*: 183 m, *Epiphaxum septifer*: 200–360 m). The discovery of *Nanipora kamurai* in this study demonstrates the importance of the study of cryptic anthozoan fauna in shallow coral reef areas (see also Fujii and Reimer 2011), not only for a more correct understanding of coral reef biodiversity, but also to make progress in clarifying the phylogeny and taxonomy of octocorals.

## Conclusions

The aragonite calcium-carbonate skeleton is considered to be a synapomorphy among Helioporacea, although only three genera including *Nanipora* are currently known from this order. Considering the close phylogenetic relationship between *Heliopora* and *Nanipora kamurai*, and the morphological affinity between *Nanipora kamurai* and *Epiphaxum*, Bayer and Muzik’s (1977) placement of *Epiphaxum* within Helioporacea based only on small surface structure and aragonite calcium-carbonate skeleton appears appropriate. In this study the phylogenetic position of *Nanipora* specimens was suggested by using a molecular phylogenetic approach. mtMutS works well in determining phylogenetic position of such unknown, unclassified species among subclass Octocorallia. Surveying cryptic environments and utilizing proper molecular markers with detailed morphological examinations are an effective way to reveal the true diversity of octocorals.

## Supplementary Material

XML Treatment for Lithotelestidae

XML Treatment for Nanipora

XML Treatment for Nanipora kamurai

## Supplementary materials

Supplementary material 1 Supplemental Table 1 Data type: species data Explanation note: List of *Nanipora kamurai* sp. n. specimens examined in this study. Collection information and GenBank accession numbers for corresponding sequences are shown. This dataset is made available under the Open Database License (http://opendatacommons.org/licenses/odbl/1.0/). The Open Database License (ODbL) is a license agreement intended to allow users to freely share, modify, and use this Dataset while maintaining this same freedom for others, provided that the original source and author(s) are credited.Yu Miyazaki, James Davis Reimer

Supplementary material 2 Supplemental Table 2 Data type: molecular data Explanation note: Outgroup sequences from GenBank used in molecular phylogenetic analyses. This dataset is made available under the Open Database License (http://opendatacommons.org/licenses/odbl/1.0/). The Open Database License (ODbL) is a license agreement intended to allow users to freely share, modify, and use this Dataset while maintaining this same freedom for others, provided that the original source and author(s) are credited.Yu Miyazaki, James Davis Reimer

Supplementary material 3 Supplemental Table 3 Data type: molecular data Explanation note: Genetic distances (p-distances) for mtMutS between *Nanipora kamurai* and *Heliopora coelurea* and for other species of octocorals included in mtMutS phylogeny (see Fig. 12 and Suppl. material 2). This dataset is made available under the Open Database License (http://opendatacommons.org/licenses/odbl/1.0/). The Open Database License (ODbL) is a license agreement intended to allow users to freely share, modify, and use this Dataset while maintaining this same freedom for others, provided that the original source and author(s) are credited.Yu Miyazaki, James Davis Reimer

Supplementary material 4 Supplemental Table 4 Data type: molecular data Explanation note: Genetic distances (p-distances) for mtMutS between *Nanipora kamurai* and *Heliopora coelurea*, and for other species of octocorals included in COI phylogeny (See Suppl. material 2). This dataset is made available under the Open Database License (http://opendatacommons.org/licenses/odbl/1.0/). The Open Database License (ODbL) is a license agreement intended to allow users to freely share, modify, and use this Dataset while maintaining this same freedom for others, provided that the original source and author(s) are credited.Yu Miyazaki, James Davis Reimer
